# Whole-genome fingerprint of the DNA methylome during chemically induced differentiation of the human AML cell line HL-60/S4

**DOI:** 10.1242/bio.044222

**Published:** 2020-02-17

**Authors:** Enoch B. Antwi, Ada Olins, Vladimir B. Teif, Matthias Bieg, Tobias Bauer, Zuguang Gu, Benedikt Brors, Roland Eils, Donald Olins, Naveed Ishaque

**Affiliations:** 1 Division of Theoretical Bioinformatics, German Cancer Research Center (DKFZ), Heidelberg, Germany; 2 Molecular and Cellular Engineering, Signalling Research Centres BIOSS and CIBSS, University of Freiburg, Freiburg, Germany; 3 Heidelberg Biosciences International Graduate School (HBIGS), Heidelberg, Germany; 4 Department of Pharmaceutical Sciences, College of Pharmacy, University of New England, Portland, ME, USA; 5 School of Life Sciences, University of Essex, Colchester, UK; 6 Heidelberg Center for Personalized Oncology (DKFZ-HIPO), German Cancer Research Center (DKFZ), Heidelberg, Germany; 7 Charité – Universitätsmedizin Berlin, corporate member of Freie Universität Berlin, Humboldt-Universität zu Berlin, and Berlin Institute of Health; 8 Digital Health Centre, Berlin Institute of Health (BIH), Anna-Louisa-Karsch-Str. 2, 10178 Berlin, Germany; 9 Division of Applied Bioinformatics, German Cancer Research Center (DKFZ), Heidelberg, Germany; 10 German Cancer Consortium (DKTK), Core Center, Heidelberg, Germany; 11 Translational Lung Research Center Heidelberg (TLRC), German Center for Lung Research (DZL), University of Heidelberg, Heidelberg, Germany

**Keywords:** DNA methylation, Promyelocyte, Differentiation, Epigenomic regulation, Long range interactions, HL60

## Abstract

Epigenomic regulation plays a vital role in cell differentiation. The leukemic HL-60/S4 [human myeloid leukemic cell line HL-60/S4 (ATCC CRL-3306)] promyelocytic cell can be easily differentiated from its undifferentiated promyelocyte state into neutrophil- and macrophage-like cell states. In this study, we present the underlying genome and epigenome architecture of HL-60/S4 through its differentiation. We performed whole-genome bisulphite sequencing of HL-60/S4 cells and their differentiated counterparts. With the support of karyotyping, we show that HL-60/S4 maintains a stable genome throughout differentiation. Analysis of differential Cytosine-phosphate-Guanine dinucleotide methylation reveals that most methylation changes occur in the macrophage-like state. Differential methylation of promoters was associated with immune-related terms. Key immune genes, *CEBPA*, *GFI1*, *MAFB* and *GATA1* showed differential expression and methylation. However, we observed the strongest enrichment of methylation changes in enhancers and CTCF binding sites, implying that methylation plays a major role in large-scale transcriptional reprogramming and chromatin reorganisation during differentiation. Correlation of differential expression and distal methylation with support from chromatin capture experiments allowed us to identify putative proximal and long-range enhancers for a number of immune cell differentiation genes, including *CEBPA* and *CCNF*. Integrating expression data, we present a model of HL-60/S4 differentiation in relation to the wider scope of myeloid differentiation.

## Abstract (precis)

**Summary:** Epigenomics play a role in cell identity and differentiation. We present the DNA methylation landscape of leukemic cells during *in vitro* differentiation, adding another omics layer to better understand differentiation mechanisms.

## INTRODUCTION

Gene expression profiles differ among different cell types and change as stem cells differentiate (Cheng et al., 1996; Le Naour et al., 2001; Natarajan et al., 2012). Genome-wide Cytosine-phosphate-Guanine dinucleotide (CpG) methylation, an epigenetic regulation and modification process, has been shown to exhibit similar dynamic behaviour during differentiation (Brunner et al., 2009; Bock et al., 2012). Usually, these two changes (i.e. gene expression and CpG methylation) have been shown to correlate negatively with each other, depending upon the location of the methylated CpG relative to the gene body (Payer and Lee, 2008; Chuang et al., 2012; Jones, 2012; Yang et al., 2014). Overall, changes in methylation patterns between cell types and tissues throughout life work to either activate or shut down specific cellular processes (Smith and Meissner, 2013), making cells exhibit different phenotypic characteristics. Acting as a shutdown mechanism, DNA methylation reinforces gene silencing, when expression is not required in a particular cell type (Lock et al., 1987).

Normal myeloid cell differentiation occurs within the bone marrow, where stroma cells secrete cytokines to help activate myeloid-specific gene transcription (De Kleer et al., 2014). Further differentiation can occur in the peripheral tissues or blood, dependent upon exposure of the myeloid precursors to cytokines and other factors, such as antigens (Álvarez-Errico et al., 2015; Geissmann et al., 2010). The first direct committed step toward myeloid cell development is the differentiation of multipotent progenitors (MPP) cells into common myeloid progenitor cells (CMP) (Kondo et al., 1997; Álvarez-Errico et al., 2015). CMP cells can then differentiate further into the granulocyte-macrophage lineage progenitor (GMP) and megakaryocyte-erythroid progenitor (MEP) (Iwasaki and Akashi, 2007). While CMP cells can differentiate into all myeloid cell types, GMP cells give rise mainly to monocytes/macrophages and neutrophils, together with a minor population of eosinophils, basophils and mast cells (Álvarez-Errico et al., 2015; Iwasaki and Akashi, 2007; Laiosa et al., 2006).

The human myeloid leukemic cell line HL-60/S4 [human myeloid leukemic cell line HL-60/S4 (ATCC CRL-3306)] is an excellent system to study epigenetic changes during chemically induced *in vitro* cell differentiation. HL-60/S4 cells are supposedly blocked at the GMP cell state and unable to differentiate any further. The HL-60/S4 cell line is a subline of HL-60 and demonstrates ‘faster’ cell differentiation than the parent HL-60 cells. Undifferentiated HL-60/S4 cells exhibit a myeloblastic or promyelocytic morphology with a rounded nucleus containing two to four nucleoli, basophilic cytoplasm and azurophilic granules (Birnie, 1988). Retinoic acid (RA) can induce HL-60/S4 differentiation to a granulocyte-like state. 12-O-tetradecanoylphorbol-13-acetate (TPA) can induce differentiation to monocyte/macrophage-like states (Birnie, 1988; Fontana et al., 1981).

**Fig. 1.**
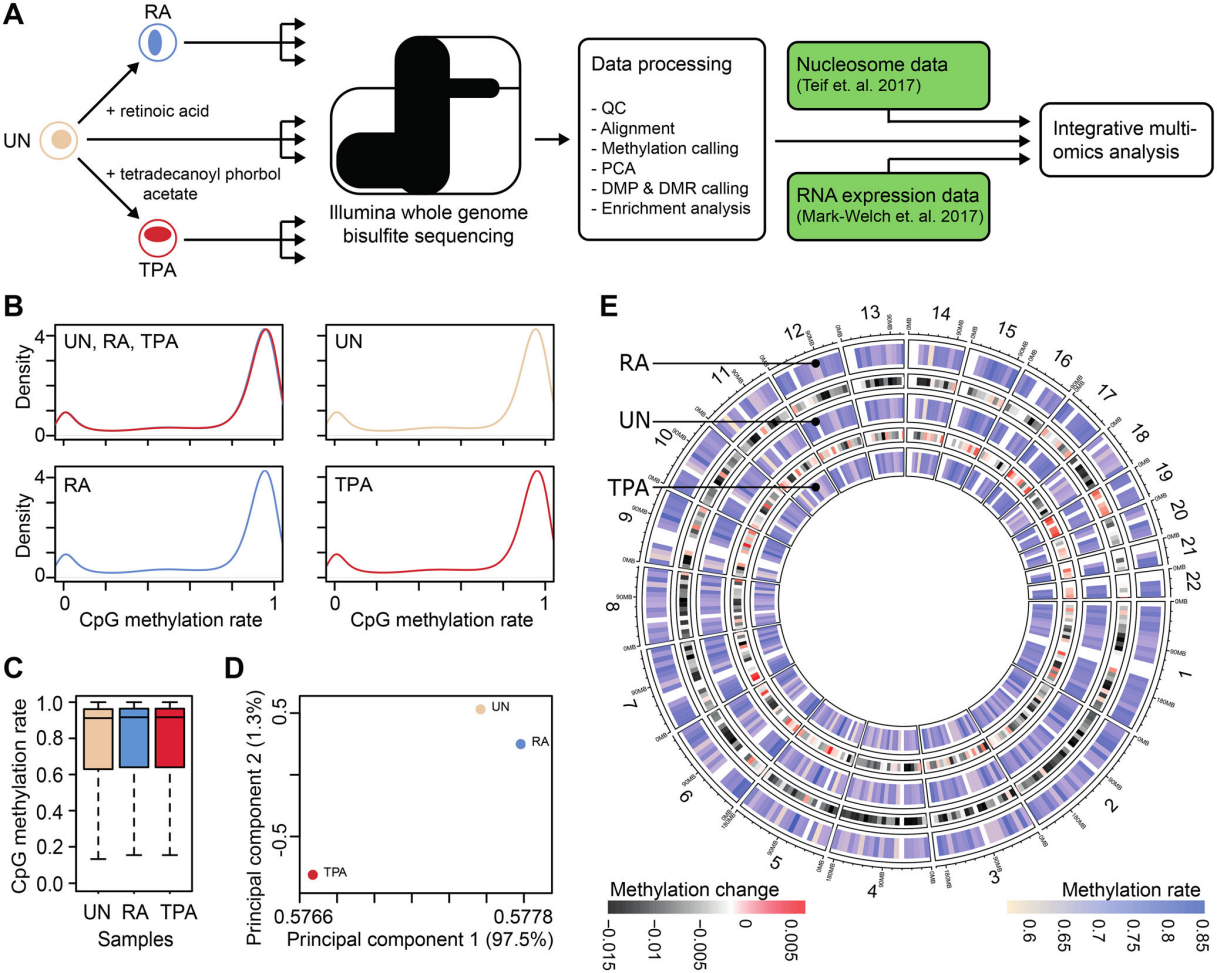
**Analysis of DNA methylome upon chemical induction of differentiation of HL-60/S4 cells.** (A) Schematic diagram of the experimental design of the study. (B) Whole-genome CpG methylation rate density plot. The upper left density plot shows that all three cell states (UN, RA and TPA) have very similar genome-wide CpG methylation rates. The subsequent density plots show the CpG methylation rates for each cell state separately. (C) Box plots summarising the distribution of CpG methylation rates per sample replicates for the ∼22 million CpGs with coverage ≥10× in all samples. The upper and lower limits of the boxes represent the first and third quartiles, respectively, and the black horizontal line is the median. The whiskers indicate the variability outside the upper and lower quartiles. (D) Principal component analysis of the WGBS data for the three cell states. Principal component 1 and 2 separate TPA from UN and RA cells. (E) Circular representation of DNA methylation rates for the different treatments. CpG methylation rates (colour scale beige–blue) were averaged over 10-Mb windows and are presented as heatmap tracks. The heatmaps show the DNA methylation change (heatmap black–white-red) with respect to the samples in the adjacent tracks.

The extent to which DNA methylation regulates these chemically induced differentiation processes is not known. Likewise, the global genome-wide methylation changes associated with these differentiation processes have not been described. This study details the methylation changes (and lack of changes), when HL-60/S4 is differentiated to granulocytes employing RA, and to macrophages employing TPA. The information contained within this study is intended as a sequel to previous studies that describe the transcriptomes (Mark Welch et al., 2017), nucleosome positioning (Teif et al., 2017) and epichromatin properties (Olins et al., 2014) of HL-60/S4 cells differentiated under identical conditions. The goal is to integrate these different lines of information into a comprehensive description and mechanistic analysis of the cell differentiation pathways in the human myeloid leukemic HL-60/S4 cell lineage. A graphical overview of our study is shown in Fig. 1A.

## RESULTS

### Little or no DNA methylation changes are observed upon HL-60/S4 cell differentiation at the megabase scale

We performed whole-genome bisulphite sequencing (WGBS) of HL-60/S4 in three different cell differentiation states: the undifferentiated state (UN), the RA-treated granulocyte state, and the TPA-treated macrophage state. Comparison of the whole- genome coverage profiles for each of the three differentiation states of HL-60/S4 revealed that the cell line is hypo-diploid (Mark Welch et al., 2017) and is chromosomally stable throughout differentiation (Fig. S1A–C). A comparison of HL-60/S4 cells (from 2008 and 2012) by fluorescent *in situ* hybridization (FISH) karyotyping showed that this cell line is also stable over long time periods (Fig. S1D,E). From all the CpGs identified by WGBS on all three cell states, a total of 21,974,649 (82.38%) CpGs had ≥10× coverage (Table 1 and Table S1), which spanned the full range of methylation rates, from 0 (completed unmethylated) to 1 (fully methylated). Most of these CpGs are highly and fully methylated (>0.75 methylation rate), with only small sets of lowly and unmethylated CpGs (<0.25 methylation rate) and partially methylated CpGs (methylation rate from 0.25 to 0.75) (Fig. 1B,C). Principal component analysis of all CpGs with coverage greater than 10 revealed that the RA-treated samples differed only slightly from the untreated sample, while the TPA samples had a much higher methylation variance compared to the other two samples (Fig. 1D). However, little or no methylation differences were observed among the three samples, when methylation rates were averaged over 10 megabase (Mb) windows (Fig. 1E).

**Table 1.**
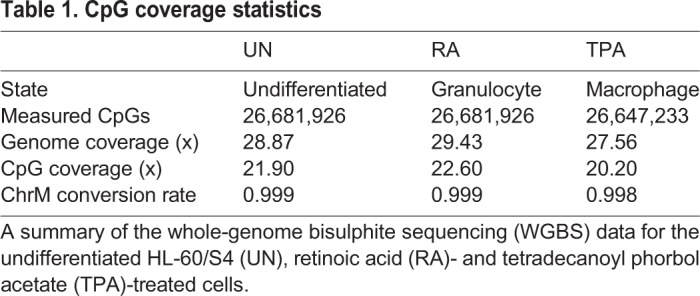
**CpG coverage statistics**

### The single CpG methylation landscape of TPA cells differ most, when compared to UN and RA cells

**Fig. 2.**
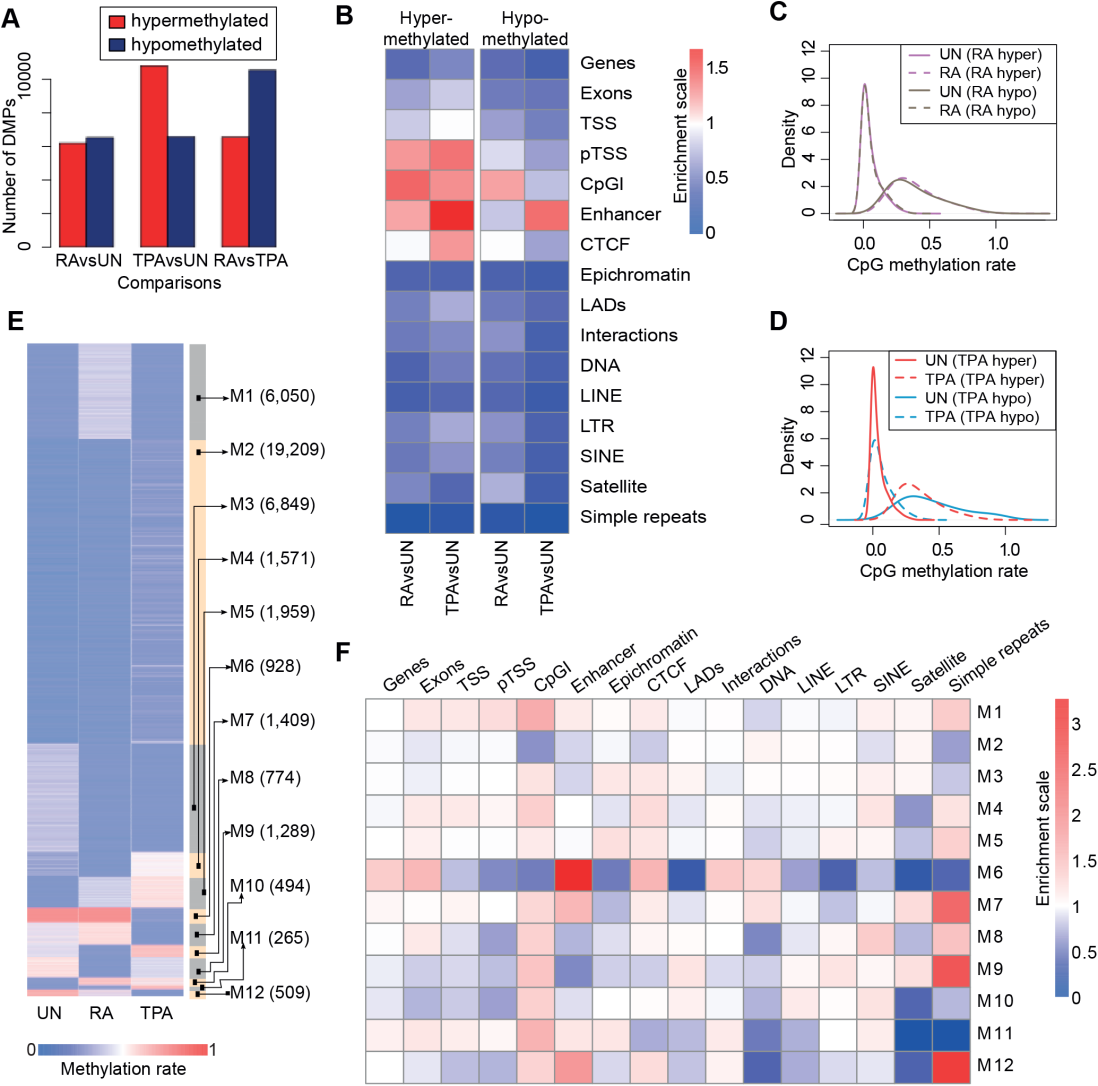
**DMP analysis.** (A) Number of DMPs identified with Fisher exact test for each comparison. In the bar plot, the x-axis labels indicate the comparisons, and the y-axis indicates the number of identified DMPs. The number of hypermethylated DMPs are shown by the red bars, and the hypomethylated by blue bars. (B) Enrichment of genomic features in the hypermethylated (left) and hypomethylated (right) DMPs in RA and TPA compared UN cells. Genes, exon and TSS features are of all genes in the gencode v19 gene models. PTSS denotes the TSS of protein coding genes. CpGI denotes CpG islands. Enhancers were identified from ENCODE chromHMM chromatin segmentation. CTCF binding sites were from ENCODE ChIP-seq experiments. Epichromatin and LADs have been identified to associate with the nuclear envelope. Integrations denote chromatin interacting regions as defined to chromatin conformation capture experiments in ENCODE. DNA, LINE, LTR, SINE, satellite and simple repeats are all classes of repeats. (C) Density plot of the methylation rates of RA DMPs in UN and RA. Hypermethylated DMPs increased from methylation rates of 0 to 0.2, and hypomethylated posited decreased from 0.2 to 0. Hyper and hypomethylated DMPs found in RA compared to UN are denoted by (RA hyper) in pink and (RA hypo) in grey respectively; the methylation rate of the DMPs in UN and RA are denoted as solid and dashed lines, respectively. (D) As panel C, but for TPA. TPA hypermethylated DMPs (TPA hyper, in red) and hypomethylated DMPs (TPA hypo, in blue). (E) Unsupervised cluster analysis reveals 12 DMP ‘modules’. 12 modules were identified, and a heatmap representation showed distinct methylation rates in each of the three cell states. Modules are demarcated by alternating grey and beige bars on the right. (F) Genomic feature enrichment in the 12 modules. Module M6 shows a strong enrichment for enhancers, but not TSS regions. Simple repeat elements are enriched in states M7, M9 and M12. The genomic features are explained in B.

Due to the small changes observed on the megabase scale, we focused on significantly differentially methylated single CpGs positions (DMPs) for further analysis. A total of 41,306 unique CpGs were identified to be significantly differentially methylated (Fisher analysis, see Materials and Methods). These DMPs comprise of 12,713, 17,392 and 17,100 CpGs from the comparisons of RA to UN cells, TPA to UN cells and RA to TPA cells, respectively (Fig. 2A). A higher proportion of the DMPs identified in the comparison of TPA to UN cells were hypermethylated; but a similar number of hyper- and hypomethylated DMPs were observed in the RA to UN cells comparison. Most of the hypermethylated DMPs (in RA to UN and TPA to UN comparisons) had a methylation rate increase from 0 to 0.2; hypomethylated DMPs showed a decrease of methylation rate (0.2–0) (Fig. 2C,D).

### Enhancers are most enriched within DMPs

We performed enrichment analysis of various genomic features representing active regulatory elements (enhancers, CpG Islands, chromatin interacting regions), gene-related features (genes, exons, transcription start sites), chromatin organisation [CTCF binding sites, laminal associated domains (LADs), epichromatin], and a number of DNA repeat elements [DNA, long interspersed nuclear element (LINE), long terminal repeats (LTR), short interspersed nuclear element (SINE), satellite and simple repeats (tandem duplications of short simple sets of DNA bases)]. The most enriched genomic features in the hypermethylated DMPs were enhancers, transcription start sites (TSSs) of protein coding genes (pTSS) and CpG islands (CpGIs) for both RA and TPA cells compared to UN cells (Fig. 2B). CTCF, a chromatin-binding protein known for its role as an insulator, regulating transcription and chromatin architecture, was enriched in TPA hypermethylated DMPs, but not in RA. On the other hand, CpGIs were also the most enriched feature in the hypomethylated DMPs, when RA was compared to UN cells. Enhancers, regulatory regions bound by transcription factors that play a cis-activating role of transcription, showed a high enrichment in both hypermethylated and hypomethylated DMPs identified when TPA is compared to UN cells (Fig. 2B). In contrast to enhancers, epichromatin (regions of chromatin that are present at the surface of chromatin in close proximity to the nuclear envelope), and both simple repeats (tandem duplications of short simple sets of DNA bases) and LINE repeats (constituting 21% of the human genome and are normally silenced) were depleted within hyper and hypomethylated regions in both RA and TPA.

We identified clusters of DMP methylation pattern changes between the three-cell states of HL-60/S4. We called these clusters ‘modules’. Module analysis reveals that enhancers are significantly enriched in DMPs that are hypomethylated in the TPA state relative to UN and RA (modules M6 and M12). Module M6 was not enriched in gene transcription start sites. The hypomethylated DMPs of TPA cells in modules M6 and M12 corresponded with lower nucleosome occupancy (Fig. S2). M7 DMPs were similarly hypomethylated in the TPA, compared to RA and UN cells, but with lower methylation differences (Fig. 2E,F). Enrichment of exons, epichromatin and chromatin-interacting domains (Teng et al., 2015) were also observed in module M6. The modules exhibited varying enriched GO functional terms (Tables S2-14).

### CpGIs have a very dynamic differential methylation

CpGIs are differentially methylated, but mainly in relation to RA-treated cells (Fig. 2B). CpGIs were most enriched in module M1, which has DMPs that are hemimethylated (∼0.5 methylation rate) in RA; but these DMPs showed lower methylation in TPA and UN cells. Similar results were seen in module M9, where DMPs were hypomethylated in RA, compared to TPA and UN cells (Fig. 2F). Likewise, CpGI enrichment was observed for module M11, where DMPs are hypermethylated in TPA, compared to RA and UN cells.

### Methylation of transcriptional activator and suppressor binding sites correlate with gene expression

**Fig. 3.**
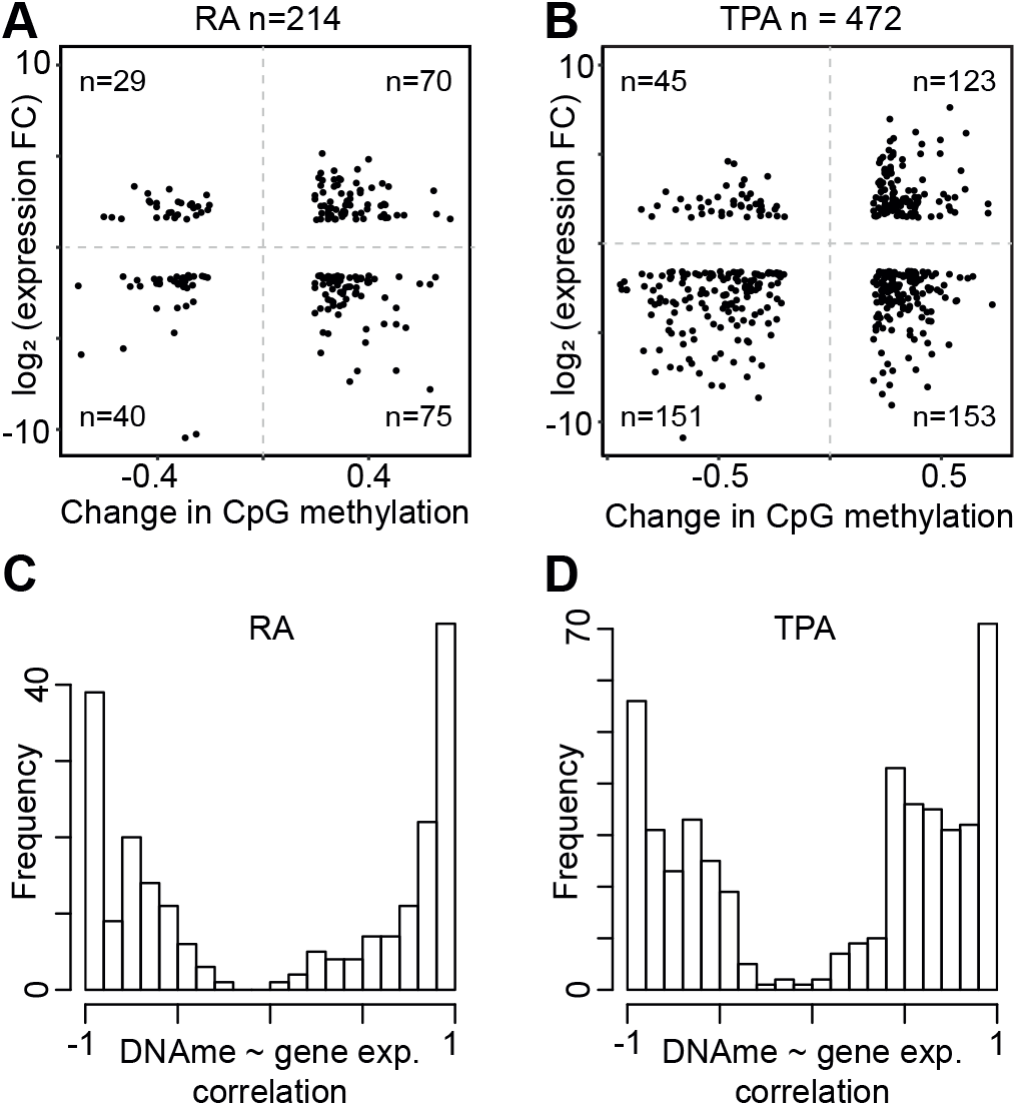
**Association between promoter methylation and gene expression.** (A) Scatter plot of genes with more than 1.5× log2 fold change in expression against the DNA methylation different (minimum of 0.2) of upstream transcription factor binding region between RA and UN cells. The number of genes is identified in the title of the plot. In each quadrant the number of genes in reported, with the upper right quadrant and lower left indicating genes with possible positive correlation with DNA methylation, and the upper left and lower right quadrants indicating genes that have negative correlation with DNA methylation. (B) As panel A, but for TPA. The scatter plots shows a comparable number of genes with increased TSS methylation with up and downregulation, however genes with reduced TSS methylation also exhibit reduced expression. (C) The distribution of Pearson correlation coefficient values for each gene between the gene expression value and the average methylation of DMPs overlapping with TSS in UN, RA and TPA cells. Increase in DNA methylation is associated with nearly as many up and downregulated genes as with decrease in methylation. While we expect to see primarily negative correlation, the histogram shows comparable numbers of highly positive and highly negative correlations. (D) As panel C, but for TPA. This histogram shows an elevated number of genes with positive correlation between 0.5 and 0.8.

DNA methylation plays a general role in inhibiting the binding of a number of transcription factors (TF) in regulatory regions of their target gene(s). In these regions one would expect a negative correlation between DNA methylation and expression, e.g. increased promoter methylation associated with decreased gene expression. To investigate the effect of DNA methylation in regulatory regions upstream of genes during HL-60/S4 differentiation, we identified all DMPs with a change in methylation rate of at least 0.2 lying in upstream regulatory regions of genes with a log2 fold change in gene expression of at least 1.5. A total of 214 and 472 DMPs were identified in RA and TPA cells compared to UN cells, respectively (Fig. 3A,B). In RA cells, genes showed no clear association of changes in gene expression and DMP methylation rate (Fig. 3A). To investigate this further, for each of these genes we performed correlation analysis of the average TSS methylation in UN, RA and TPAs with the gene expression of the gene in UN, RA and TPA cells. This showed a comparable number of positive correlating genes (+1) and negatively correlating genes (−1) (Fig. 3C). This may explain the unclear association observed in Fig. 3A. However, TPA cells showed a higher number of positive associations for hypomethylated DMPs (Fig. 3B). Correlating the average TSS methylation and gene expression for these genes showed a higher number of positively correlating genes (in the range of 0.5–0.8), compared to negatively correlating ones (Fig. 3B,D). This positive correlation may be explained by DNA methylation preventing the binding of transcriptional suppressors, therefore increasing the expression of target genes. We found that genes associated with regulatory regions which displayed positive correlation (1.0–0.7) between DNA methylation and expression were significantly enriched in EZH2 binding (q-value of 0.087 and 0.030 in RA and TPA, respectively) but not those with negative correlation (q-value of 0.197 and 1 in RA and TPA, respectively). EZH2 is a well-known transcriptional repressor that forms part of the polycomb repressor complex 2. DNA methylation of EZH2 binding sites would therefore have a positive effect on transcription, explaining the observed positive correlation of gene expression and DNA methylation.

### Methylation of long distance regulatory regions shows negative correlation with target gene expression

**Fig. 4.**
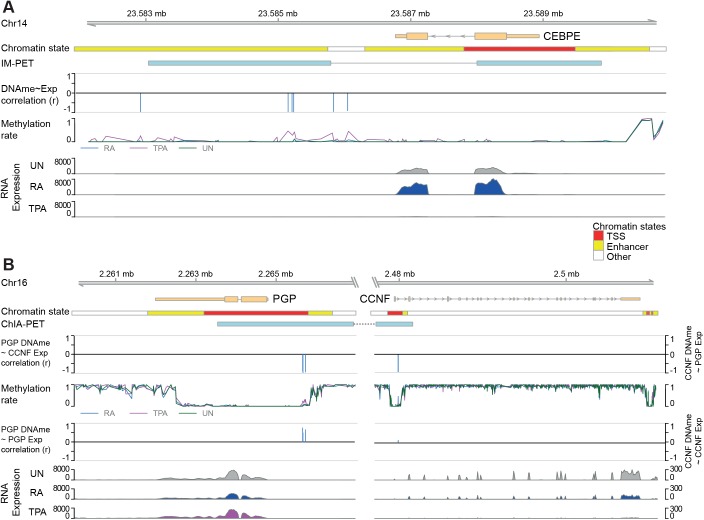
**Chromatin interacting regions explain gene expression correlation with distal DMPs.** (A) CEBPE shows a strong inverse correlation between its expression and methylation of DMPs in its downstream region. Tracks (from top to bottom): genomic coordinates; gene model of CEBPE; simplified chromatin segmentation based on E029 (primary monocyte cells from peripheral blood) from the ROADMAP epigenome project; chromatin interacting regions (K562 cells from the ENCODE project using IM-PET); bar plot depicts correlations of DMPs with CEBPE differential gene expression, indicating a number of downstream DMPS negatively correlating with CEBPE expression (where a bar below 0 indicates negative correlation and a bar above 0 indicates positive correlation between DNA methylation and gene expression); line plot of DNA methylation rate of CpGs in UN, RA and TPA cells; coverage plot of RNAseq expression in UN, RA and TPA cells. (B) The cyclin-F-box protein coding gene CCNF interacts with a distant upstream region, which regulated its expression through methylation. Panel description is similar to A, except that the regions between PGP and CCNF are cut, the CHIA-PET (from K562 cells from the ENCODE project) interaction between the upstream of PGP and CCNF are connected, the top DMP-gene expression correlation panel is of proximal CpGs with the distal gene (‘PGP DNAme∼CCNF Exp’ and ‘CCNF DNAme∼PGP Exp’) and the lower correlation panel is of proximal CpGs with the proximal gene (‘PGP DNAme∼PGP Exp’ and ‘CCNF DNAme∼CCNF Exp’). For CCNF, no proximal DMP correlated highly with CCNF expression (right track ‘CCNF DNAme∼CCNF Exp’), however a number of DMPs upstream of PGP (in a region interacting with the CCNF promoter in K562 cells) exhibited negative correlation with CCNF differential expression (right track ‘CCNF DNAme∼PGP Exp’). Together, this could imply that these DMPs upstream of PGP play a role in CCNF expression. Please note that the chromatin interaction data are derived from different cells than the chromatin state data (K562 and primary monocytes respectively). While both are derived from myeloid cells, they are different from our myeloid HL-60/S4 cells.

DMP methylation and expression of *CEBPE* (a major transcription factor involved in myeloid cell differentiation) shows a negative correlation at the 3′ end of the gene; a region identified to be an enhancer in the ROADMAP epigenome project (Fig. 4A) (Kundaje et al., 2015). The downstream region of the *CEBPE* gene, containing the DMPs, the methylation of which has strong negative correlation with expression, has been shown through IM-PET (integrated method for predicted enhancer targets) and CHIA-PET (chromatin interaction analysis by paired-end tag sequencing) to interact with the upstream regions that span part of the gene body and the TSS region (Teng et al., 2015). No DMPs were observed overlapping the TSS of the *CEBPE* gene; hence, no correlation between TSS methylation and expression is available. The RNA expression tracks of *CEBPE* (as well as *CCNF* and *PGP*) in UN, RA and TPA is shown in Fig. 4A (and 4B).

Furthermore, the RNA expression of the gene encoding for cyclin F, *CCNF*, has weak positive correlation with the methylation of DMPs that overlap with its gene and TSS (Fig. 4B, right track ‘*CCNF* DNAme∼*CCNF* Exp’). However, *CCNF* RNA expression has a strong negative correlation with DMPs overlapping the upstream region of the PGP gene (Fig. 4B, left track ‘*PGP* DNAme∼*CCNF* Exp’), which encodes phosphoglycolate phosphatase. *PGP* RNA expression does not show a similar correlation to its upstream DMPs (Fig. 4B, right track ‘*PGP* DNAme∼*PGP* Exp’). This region has also been identified by ROADMAP as an enhancer and to interact with the promoter of *CCNF* in other myeloid cells (K562 and monocytes).

### Functional annotation of DMPs are mostly immune-response-related

Using DMPs with a methylation rate change ≥0.2 we observed that immune-response-related cellular functions were the most enriched biological function for all the genes whose TSS overlapped with DMPs when RA cells were compared to UN cells (Table 2). Similarly, genes with their TSS overlapping DMPs in TPA compared to UN cells, were also mostly related to (or involved with) phosphoproteins, signalling and defence responses, including chemotaxis (Table 3). Similar observations were made when DMPs were merged into differentially methylated CpG regions (DMRs) and their functional associations tested in TPA compared to UN cells (Table 4). For RA compared to UN cells, the functional annotation was general-cell-function-related (Table 5).

**Table 2.**
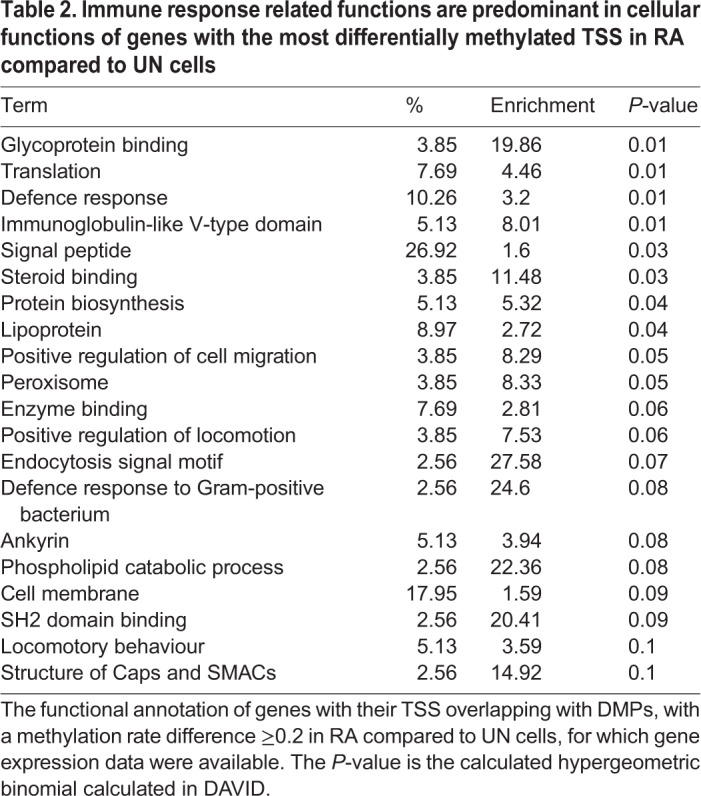
**Immune response related functions are predominant in cellular functions of genes with the most differentially methylated TSS in RA compared to UN cells**

**Table 3.**
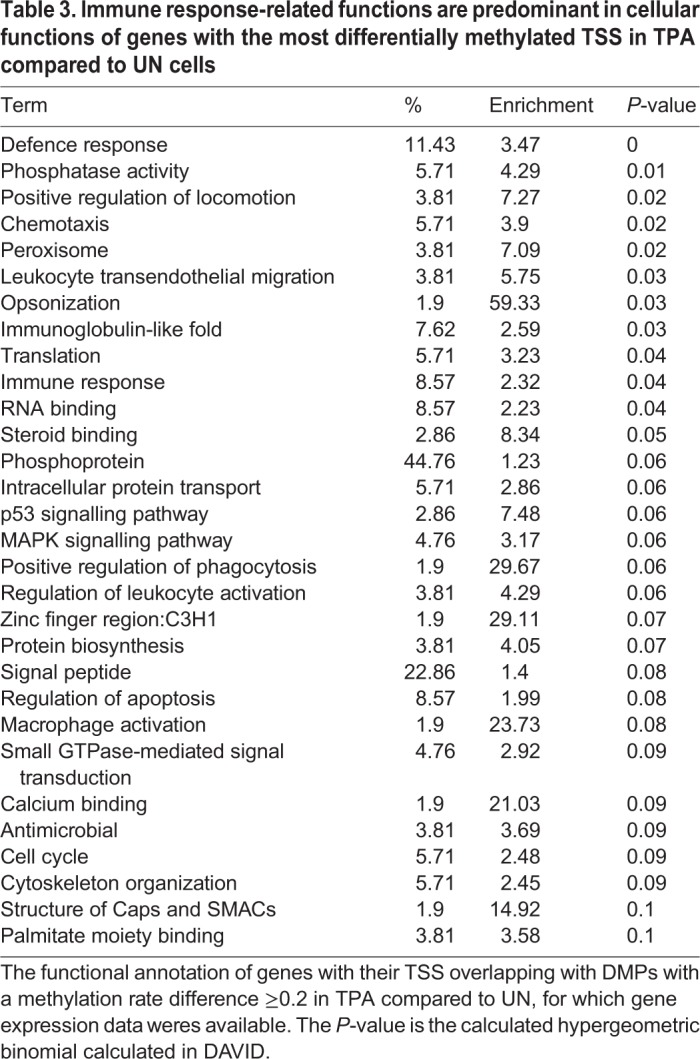
**Immune response-related functions are predominant in cellular functions of genes with the most differentially methylated TSS in TPA compared to UN cells**

**Table 4.**
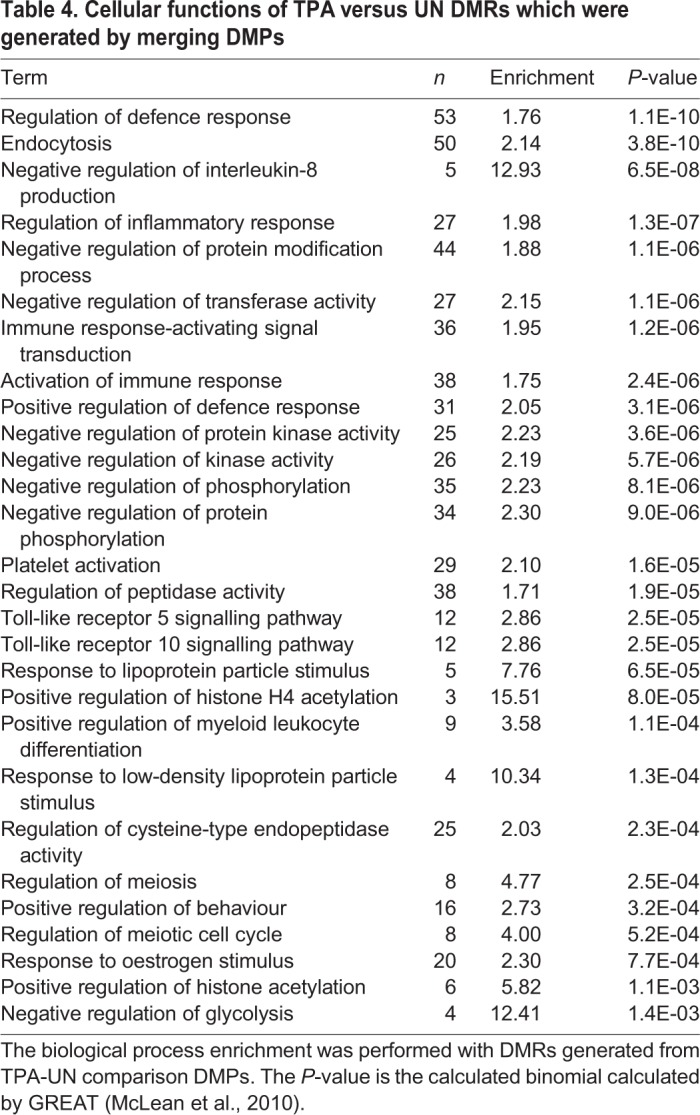
**Cellular functions of TPA versus UN DMRs which were generated by merging DMPs**

**Table 5.**
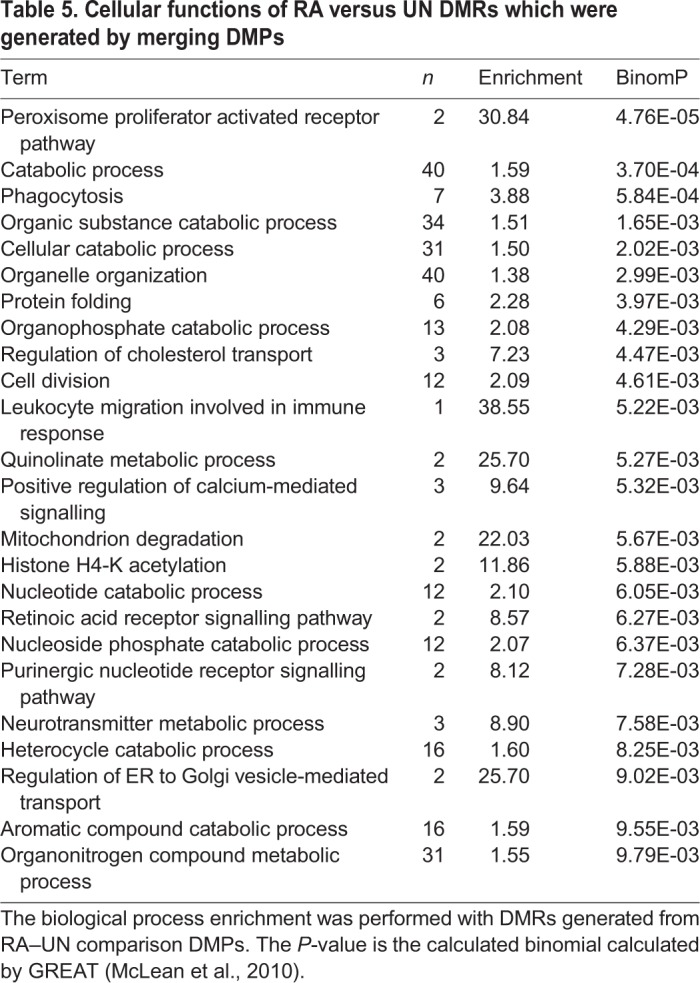
**Cellular functions of RA versus UN DMRs which were generated by merging DMPs**

### Key myeloid differentiation transcription factors are differentially expressed

Next, we analysed the expression and methylation profiles of important myeloid differentiation regulatory transcriptions factors SPI1, CEBPB, CEBPE, CREBBP, CEBPA, GATA1, MAFB, DNMTs and HDACs. It was observed that CEBPA (Fig. S3A,B; Table 6) and GFI1 (Fig. S3C,D) may be required to maintain HL-60/S4 in the undifferentiated state. *CEBPA* expression could be linked to DNA methylation with the expected negative correlation at two DMP positions, and *GFI1* expression could be linked to DNA methylation with negative correlation at multiple DMPs. As such, downregulation of *CEBPA* may play a role during further differentiation of HL-60/S4 to either the neutrophil-like or macrophage-like state. Meanwhile, *SPI1* and *CEBPB* are upregulated in both differentiated states (Fig. S3E,F,K,L). *CEBPB* increased expression did not correlate with DNA methylation, suggesting that it is regulated via other epigenetic mechanisms, and the increased expression of *SPI1* negatively correlates with DNA methylation at a single DMP position.

**Table 6.**
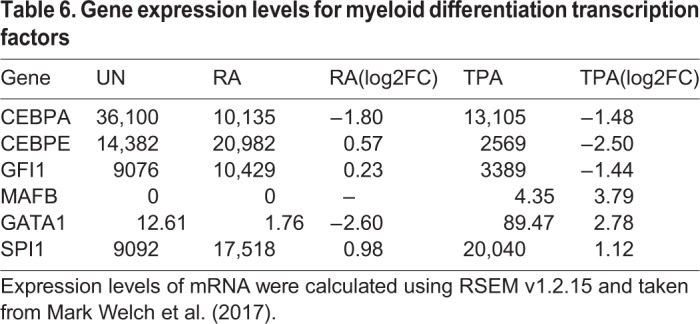
**Gene expression levels for myeloid differentiation transcription factors**

Upregulation of *CEBPE* (Fig. 4B) is seen in RA, albeit without reduced DNA methylation, whereas it is downregulated in TPA, which can be seen with increases in DNA methylation in TPA cells. The expression of *CEBPE* negatively correlated with the DNA methylation of a number of downstream DMPs. In TPA-treated cells, *MAFB* is upregulated, without correlating to changes in DNA methylation, although still at low levels (Fig. S3G,H). *GATA1* is also downregulated in RA and upregulated in TPA-treated cells without correlation to changes in DNA methylation (Fig. S3I,J).

## DISCUSSION

Understanding the molecular mechanisms that govern transcriptional reprogramming in cellular differentiation is of paramount importance to elucidate the regulatory dynamics behind this process. The realization of low-cost high-throughput sequencing methodologies have facilitated a plethora of studies outlining the landscape of transcriptional activity, regulation, chromatin organization and the complexity of which these ‘omics’ layers interact. Here we report our findings on the dynamics of DNA methylation in HL-60/S4 cells through three cell states, and perform multi-omics integrations, contextualizing our results with changes in transcription, chromatin organization, DNA features and nucleosome positioning.

We observed that the majority of the CpGs in all three cell states of HL-60/S4 were fully methylated, but did not observe large-scale DNA methylation changes during the differentiation, consistent with previous reports (Farlik et al., 2016; Kulis et al., 2015). Nevertheless, principal component analysis revealed the DNA methylation profiles of RA cells to be more similar to undifferentiated HL-60/S4 cells compared to TPA cells (Fig. 1C), supporting previous studies where neutrophil methylation was shown to be only slightly, but significantly, different from the promyelocyte precursor cell methylation (Álvarez-Errico et al., 2015). We were unable to identify any enriched GO terms from the top 1000 or 5000 CpGs contributing to principal component 1 (explaining over 97% of variation), which leads us to believe that that the changes in DNA methylation in TPA cells results in the alteration of not one specific biological process, but rather in the overall increased levels of hypermethyation in differentially methylated CpGs (Fig. 2B).

A total of 41,306 CpGs were identified to be differentially methylated (DMPs). Despite the small shifts in methylation rate (Fig. 2C,D), we observed that hypermethylated regions in RA and TPA cells were enriched for regulatory regions and CTCF binding sites, implicating a role of DNA methylation in transcriptional reprogramming and chromatin organization during differentiation. This is consistent with previous reports of a gain of methylation at CTCF binding sites in differentiated hematopoietic states compared to HSCs (Farlik et al., 2016). CTCF plays a well-known role in chromatin organization by forming loop domains involved in bringing together enhancers and promoters and topological associated domain formation (Rowley and Corces, 2018). Interestingly, the enrichment of CTCF was observed only in hypermethylated positions in TPA cells. This increased methylation of CTCF binding sites in TPA cells could be consequent of the downregulation of *CTCF* transcript NM_001191022, which showed a log2 fold reduction of 6.1 in TPA compared to UN cells (Mark Welch et al., 2017). This was also observed in a number of other human leukemic cell lines that were differentiated into monocyte like cells using TPA (Delgado et al., 1999).

The DMPs exhibited 12 distinct patterns, which we grouped into modules. While the majority of modules exhibited similar enrichment patterns to when they were simply groups of hyper and hypomethylated CpGs, module M6 stood out as being even more enriched for enhancers; simple DNA repeat elements also became enriched in a number of modules.

Module M6 has full methylation of CpGs in UN and RA but hypomethylation in TPA, suggesting a role of enhancer hypomethylation in macrophage-like differentiation, as observed in TPA-treated cells. Module M6 also showed hypomethylation of epichromatin and chromatin interaction domains in TPA cells, suggesting a remodelling of the transcriptional regulatory circuits in this state, compared to the RA and UN cell states. The hypomethylation of TPA cells in module M6 was associated with lower nucleosome occupancy (Fig. 2E; Fig. S2). Lower levels of differential methylation, differential nucleosome occupancy and enrichment for enhancers were observed for modules M7 and M12. This suggests that differential nucleosome occupancy that is associated with differential DNA methylation in our differentiation system occurs in the genomic context of enhancers. This is consistent with previous findings of changes of nucleosome occupancy and DNA methylation in regulatory genomics contexts of CTCF binding and promoters (Kelly et al., 2012) during cellular differentiation.

Modules showing the highest enrichment of simple repeats (M7, M8 and M9) exhibited hypomethylation in RA and TPA cells, but not UN cells. Hypomethylation of simple repeats has been associated with a number of diseases, including Wilms tumour and breast cancer, possibly due to the hypomethylation of these repeats predispose them to illegitimate recombination (Wilson et al., 2007). However, the biological impact of this observation is still not entirely clear.

Earlier reports suggested that methylation in the promoter and the first exon negatively correlated with gene expression (Jones, 2012; Brenet et al., 2011). However, we did not observe any general association of methylation and gene expression when comparing RA to UN cells, and only a slight but positive association for hypomethylated DMPs when comparing TPA to UN cells (Fig. 3A,B). This observation suggests that there are additional epigenetic modifications required at gene promoters regulating transcriptional activity (Ford et al., 2017 preprint) or that gene expression is determined by the epigenetic state of multiple regulatory elements and not just the promoter (Ong and Corces, 2011). When correlating the average promoter methylation of differentially expressed genes with a DMP in their promoter, we see a tendency of both highly positive (+1) and highly negative (correlation), suggesting that promoter regions comprise of both transcriptional activators as well as repressors. Indeed, we found that the gene targets of positive correlating regions were significantly enriched for binding of the transcriptional repressor EZH2, but not for the negative correlating gene targets. This highlights the importance of considering the context of regulatory regions when investigating the effects of DNA methylation on transcription.

*CCNF* expression was significantly reduced in TPA cells, compared to both UN (log2 fold reduction of 2.2) and RA cells (log2 fold reduction of 1.9) (Mark Welch et al., 2017). CCNF plays an important role during the cell cycle (Bai et al., 1994), and its downregulation in TPA cells may explain their reduced proliferation rate (Trayner and Clemens, 1992). However, we did not observe any differential methylation in its promoter that could explain this differential expression of *CCNF*. Interestingly, a region within the promoter of *PGP* contained DMPs correlating negatively with *CCNF* expression (Fig. 4B). The promoter of *CCNF* and the DMP region of *PGP* have previously been shown to physically interact, employing CHIA-PET in the K562 leukaemia cell line (The ENCODE Project Consortium et al., 2012). This finding illustrates one of the biological consequences linked to our observation of enrichment of chromatin interacting regions in DMPs.

Investigating TSS methylation and RNA expression of key myeloid differentiation transcription factors revealed that CEBPA exhibited both TSS hypermethylation and downregulation in RA and TPA compared to UN cells (Fig. S3). *CEBPA* and *SPI1* are known to be required for the maintenance of CMP and GMP developmental stages of myeloid cells (Álvarez-Errico et al., 2015). However, it is the counter-interaction between *SPI1* and CEBPA that determines whether a GMP differentiates (Iwasaki and Akashi, 2007), since *CEBPA* is known to repress macrophage differentiation induced by *SPI1*.

**Fig. 5.**
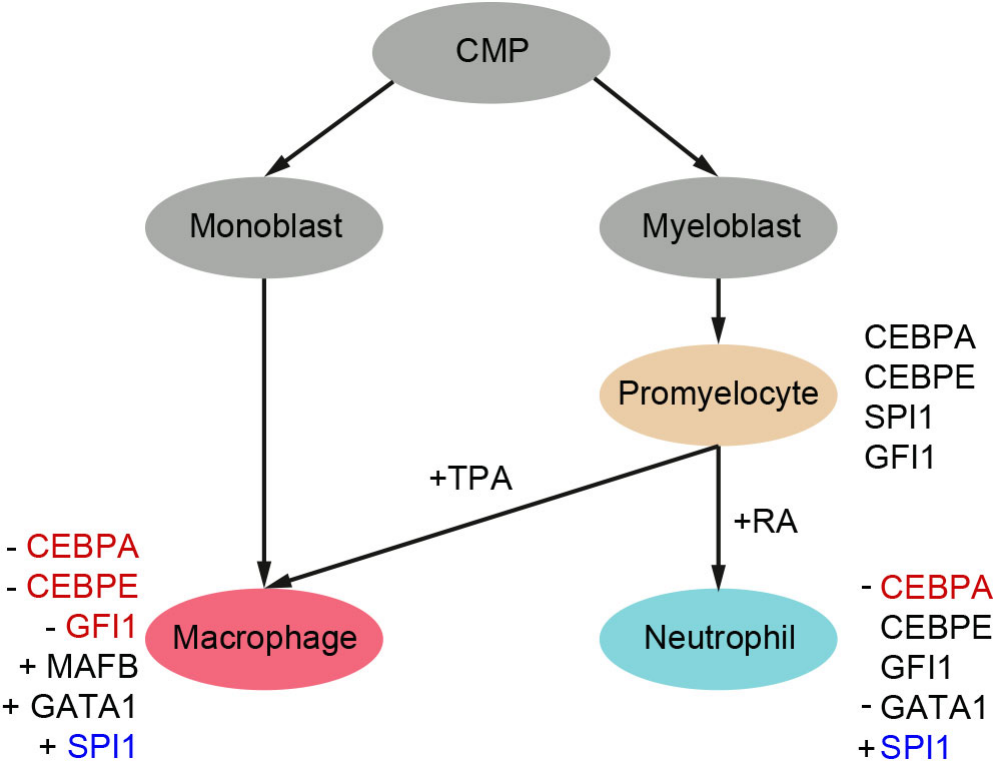
**Chemical differentiation model of HL-60/S4 showing the transcription factors that may play an essential role in determining cell fate.** Downregulation or upregulation of gene expression are denoted by ‘−’ or ‘+’ respectively. Genes with no sign attached show that their levels are maintained at similar levels as in UN (promyelocytic) state. Genes with associated DMPs either being increased or decreased in DNA methylation are coloured as red and blue, respectively.

Employing this observation, together with the data of Fig. 4, Table 6 and Fig. S3, we propose a model of the HL-60/S4 differentiation program based upon the transcription factors that may be involved (Fig. 5). In this model, we propose that downregulation of CEBPA by increased DNA methylation may be required for differentiation of HL-60/S4 cells. While *CEBPE* is highly expressed in the undifferentiated and neutrophil-like state, its downregulation due to increased DNA methylation, along with upregulation of *MAFB* and *GATA1* may be required for macrophage-like differentiation. This supports prior reports that *CEBPE* is necessary for the commitment of HL-60/S4 cells to a neutrophil-like state. It is interesting to note that all transcription factors in this model apart from *MAFB* and *GATA1* exhibited DMPs that correlated with their gene expression. This would suggest that the differential expression of *MAFB* and *GATA1* in this cell system is regulated via a different epigenetic mechanism.

Our study highlights that the major changes of DNA methylation upon HL-60/S4 cell differentiation occur at enhancers, long-range interacting regions and CTCF binding sites.

## MATERIALS AND METHODS

### Samples

We used the human AML (acute myeloid leukaemia) cell line HL-60/S4, available from ATCC (CRL-3306). Differentiation of this cell line was induced with RA and 12-O-tetradecanoylphorbol-13-acetate (TPA) to attain the granulocyte-like and macrophage-like states as previously described (Mark Welch et al., 2017). In previous publications (Teif et al., 2017; Mark Welch et al., 2017), the undifferentiated HL-60/S4 cells were denoted ‘0’. In the current study the same undifferentiated cells are denoted ‘UN’.

Phenotypic changes during HL-60/S4 differentiation are dramatic and easily observed in the microscope (Olins et al., 1998, 2000; Mark Welch et al., 2017). The rapidly dividing undifferentiated cell forms grow in suspension and display a ‘spherical’ cell shape and a ‘round’ nucleus. After RA treatment for 4 days, many of the cells weakly attach to the bottom of the growth container. Some cells show an elongated shape and appear to migrate very slowly. The nuclei lobulate, resembling normal blood granulocytes. Cell division of RA-treated cells gradually slows during the 4 day period. After TPA treatment, by 1 day many of the cells firmly attach to the substrate. Cell division has ceased. They form cell clumps that gradually recruit more cells during the ensuing days. The nucleus of TPA-treated cells remains generally ‘round’, with no indication of lobulation. RA-treated cells begin to show signs of apoptosis by days 4–5; attached TPA cells show essentially no indication of apoptosis by days 4–5. Comparison of the transcriptome data (after 4 days) from undifferentiated, RA- and TPA-treated cells show clear mRNA level changes that correlate and help to interpret the cell phenotypes observed microscopically.

### Sequencing and library preparation

WGBS libraries were prepared for UN, RA- and TPA-treated HL-60/S4 cells. Libraries were prepared using the Illumina TruSeq DNA Sample Preparation Kit v2-set A (Illumina Inc., San Diego, CA, USA) according to the manufacturer’s guidelines. After the adapters were ligated to the library, they were treated with bisulphite followed by PCR amplification. Sequencing was performed on the Illumina HiSeq 2000 using paired end mode with 101 cycles using standard Illumina protocols and the 200 cycle TruSeq SBS Kit v3 (Illumina Inc., San Diego, CA, USA). Sequencing was performed on one biological replicate, with three technical replicates.

### Read alignment and methylation calling with BSMAP

WGBS sequencing data were analysed using BsMAP (Xi and Li, 2009) and BisSNP packages. In brief, technical replicates were combined, and sequencing reads were adaptor-trimmed using CUTADAPT package (Martin, 2011), while read alignments were performed against the human reference genome (hg19 GRCh37 version hs37d5-lambda, 1000 Genomes) using the BsMAP-2.89 package with non-default parameter–v 8 (Xi and Li, 2009). Putative PCR duplicates were filtered using Picard [version 1.61(1094) MarkDuplicates (http://picard.sourceforge.net)]. Only properly paired or singleton reads with a minimum mapping quality score of ≥30 and bases with a Phred-scaled quality score of ≥10 were considered in methylation calling using the BisulfiteGenotyper command. BisulfiteTableRecalibration was called with −maxQ 40. Methylation calling was done with BisSNP package (Liu et al., 2012) and single-base-pair methylation rates (b-values) were determined by quantifying evidence for methylated (unconverted) and unmethylated (converted) cytosines at all CpG positions. Non-conversion rates were estimated using data from mitochondrion DNA (chrM). Only CpGs with coverage ≥10× in all sample replicates were considered in downstream analysis.

### Differentially methylated CpGs calling

Fisher exact test with α=0.05 was applied to all 17,233,911 CpGs individually to extract DMPs.

### Nucleosome occupancy analysis

We used publicly available nucleosome occupancy data (Teif et al., 2017), where they investigated nucleosome repositioning during differentiation of the HL-60/S4 cell line. They aligned the reads to the human genome using Bowtie (Langmead et al., 2009) (allowing one mismatch, and only considering unique alignments), after which nucTools (Vainshtein et al., 2017) was used to generate the average nucleosome occupancy profiles of ±1 kb around the CpGs within each of the identified modules, separately for each of the differentiated states of HL-60/S4.

### Principal component analysis

Principal component analysis was done on all 17,233,911 CpGs using the princomp command in R.

### Genomic features analysis

We extracted genic features (intron, exons, intergenic regions, genes TSS) together with 4D genomic interaction data from gencode v17 (Harrow et al., 2012), CpG Island, Laminal Associated Domains (LADS) and RepeatMasker definitions from UCSC (Rosenbloom et al., 2012). Using the start and end coordinates of the genes from Genecode17, TSS was defined as the region extending 2 kb upstream and 1 kb downstream the start of the gene. RepeatMaskers considered in the enrichment analysis are: DNA repeat elements (DNA), LINE, low complexity repeats, LTR, rolling circle repeats (RC), RNA repeats (RNA, rRNA, scRNA, snRNA, srpRNA and tRNA), satellite repeats, simple repeats (micro-satellites) and SINE. Enhancers were extracted from ENCODE (The ENCODE Project Consortium et al., 2012), FANTOM5 (Andersson et al., 2014) and Vista (Visel et al., 2006). Coordinates of HL-60/S4 epichromatin are described (Olins et al., 2014).

### DMP enrichment analysis

Genomic feature and chromosome enrichment in the DMPs were estimated using the formula:
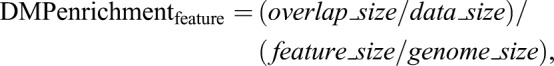
where ‘*data_size*’ is the size of the data (for either RA or TPA DMPs) used to calculate the enrichment. Note that the enrichment of the hyper and hypomethylated DMPs were calculated relative to the ‘*data_size*’ or the total DMPs or DMRs called for each comparison but not relative to the total of only hyper or hypomethylated DMPs or DMRs.

### Functional annotation

DMR functional annotations were performed with DAVID 6.8 (Huang et al., 2009) using the full set of human genes as the background.

### Differential methylation patterns of DMPs analysis

DMPs were clustered using the hclust (Murtagh, 1985) with the complete linkage method after the Euclidean distances were calculated using the dist function in R. The hierarchically clustered DMPs were divided into 12 clusters using cutree. The resulting clusters were named as modules, from module M1 to module M12.

Feature enrichment within modules were estimated using the following formula:
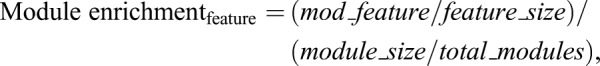
where ‘*mod_feature*’ is the size of a module overlapping with a specific genomic feature and ‘*feature_size*’ is the total size of a genomic feature in all modules. ‘*module_size*’ is the total size of a module and ‘*total_modules*’ is the size of the all modules together.

### Extraction of DMRs

DMR calling was done by first averaging coverage and number of methylation calls in three CpGs sliding windows with maximum size of 2 kb. Fisher exact testing was done using an alpha value of 0.05 to extract differentially methylated windows. Continuous differentially methylated windows were merged into one and a Fisher test with the same conditions was applied the second time to ensure the regions were significantly differentially methylated. Differentially methylated regions that had three CpGs /1 kb ratio were extracted before applying the final filter, which states that a DMR should consist of at least three sliding windows. This step was to eliminate regions that probably had only one truly differentially methylated CpG. As such, DMRs that were made of less than three windows (five CpGs) were dropped.

### Differential gene expression

Differentially expressed genes data estimated using the RSEM software package (Li and Dewey, 2011) were obtained from our collaborators in The Josephine Bay Paul Center for Comparative Molecular Biology and Evolution (USA) (Mark Welch et al., 2017). Briefly, they followed the RSEM workflow outlined at http://deweylab.github.io/RSEM using gene models from the UCSC human genome v19 available from Illumina iGenome. Bowtie2 was used to align reads to transcript models, followed by quantification using RSEM v1.2.5 using the ng-vector option for isoform analysis.

### Correlation between gene expression and TSS methylation of HL-60/S4 genes

Methylation and transcriptome data were integrated by first extracting genes with log_2_ fold change in gene expression greater than 1.5 and TSS overlapping with at least one DMP as extracted using the Fisher exact test. Secondly, we extracted DMPs with methylation rate difference greater than 0.2 that overlapped known transcription factor binding sites determined by ENCODE (http://hgdownload.soe.ucsc.edu/goldenPath/hg19/encodeDCC/wgEncodeRegTfbsClustered/wgEncodeRegTfbsClusteredV3.bed.gz). In this extraction criterion 214 and 472 genes were identified for RA and TPA cells respectively. The association between methylation change of DMPs overlapping with an upstream transcription factor binding region of a gene and the log_2_ fold change in gene expression were depicted for RA and TPA genes as a scatter plot. For each of these 214 and 472 genes, subsequent correlation analysis using the DMP methylation rate and gene expression value for UN, RA and TPA. These correlation values were reported as a histogram (Fig. 3B,D).

To investigate enrichment of transcription factor binding in positively and negatively correlating genes, we extracted the gene lists of positively (correlation of 1 to 0.7) and negatively correlating (correlation of −1.0 to −0.7) genes. Genes beginning with ‘trans_’ were removed, and hyphenated genes were separated into two. The gene lists were provided to the enrichr webtool (https://amp.pharm.mssm.edu/Enrichr/) and transcription factor enrichment was determined from the ‘ENCODE TF ChIP-seq 2015’ results.

Furthermore, the correlation between the methylation of individual CpGs in the gene body and TSS region and gene expression was estimated for all genes from both extraction criteria together with the gene expression of transcription factors known to be involved in myeloid cell differentiation (Fig. 5).

## Supplementary Material

Supplementary information
