# Supplementary material for: Whole-genome fingerprint of the DNA methylome during chemically induced differentiation of the human AML cell line HL-60/S4
Source: Biol Open. 2020 Feb 17;9(2):bio044222. doi: 10.1242/bio.044222 (PMC7044446; doi:10.1242/bio.044222)
Supplement: Supplementary information [file biolopen-9-044222-s1.pdf]

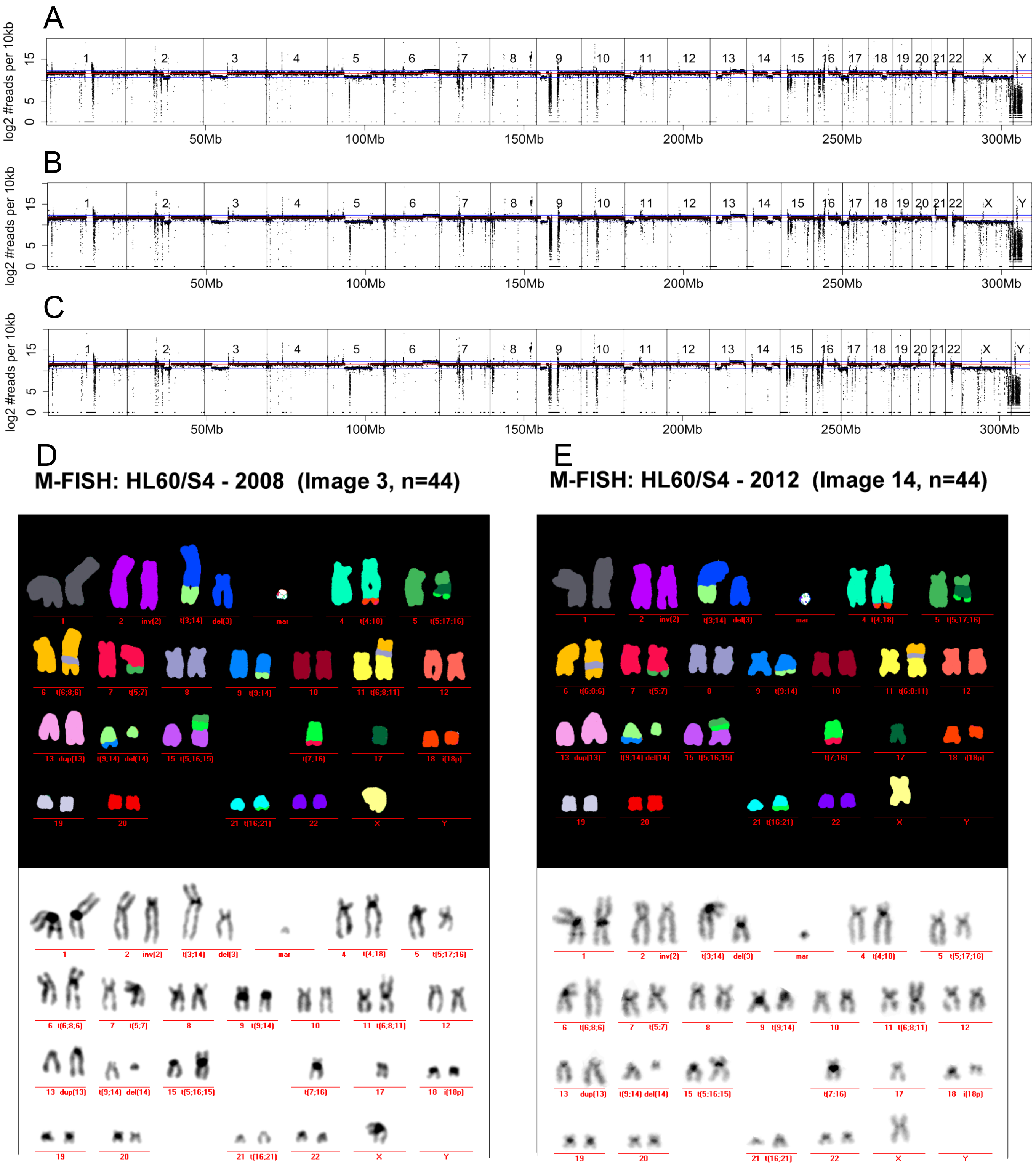

Figure S1

Figure S1: Genome of HL60/S4 is stable over time and upon differentiation: Coverage plots of the WGBS data for UN (A), RA (B), and TPA cells (C) depicting stable genome during differentiation. D and E show 2 examples of M-FISH of undifferentiated HL-60/S4 over a period of 4 years depicting stability of the genome.

Biology Open • Supplementary information

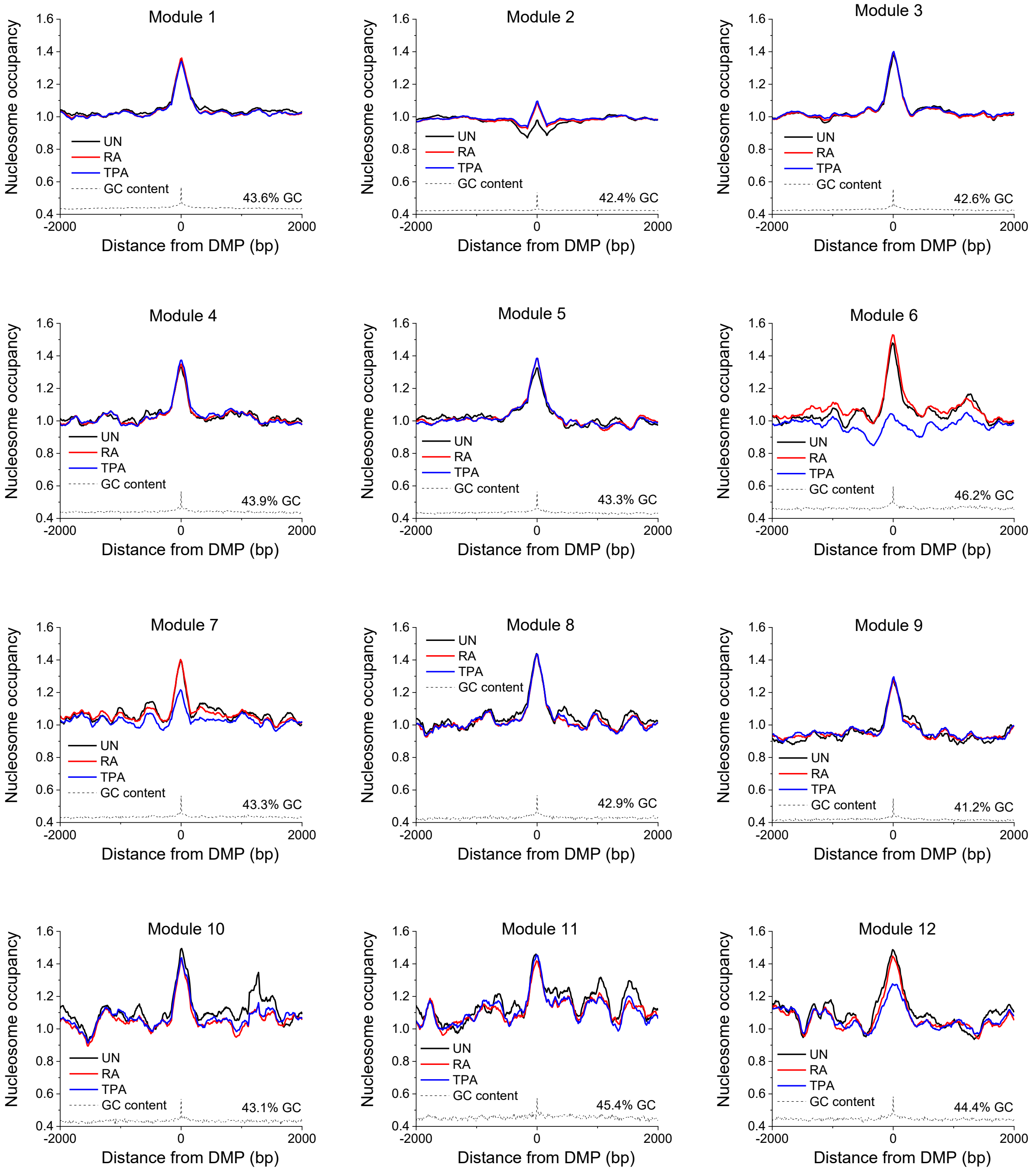

Figure S2

Figure S2: Average nucleosome occupancy around DMP of the different modules as described in figure 2. Each image shows nucleosome occupancy 2000 bases up- and downstream of DMPs per module. Nucleosome occupancy is shown in black, red and blue for untreated, RA and TPA treated respectively. GC content refers to the percentage of GC at each base relative to the DMP position.

Biology Open • Supplementary information

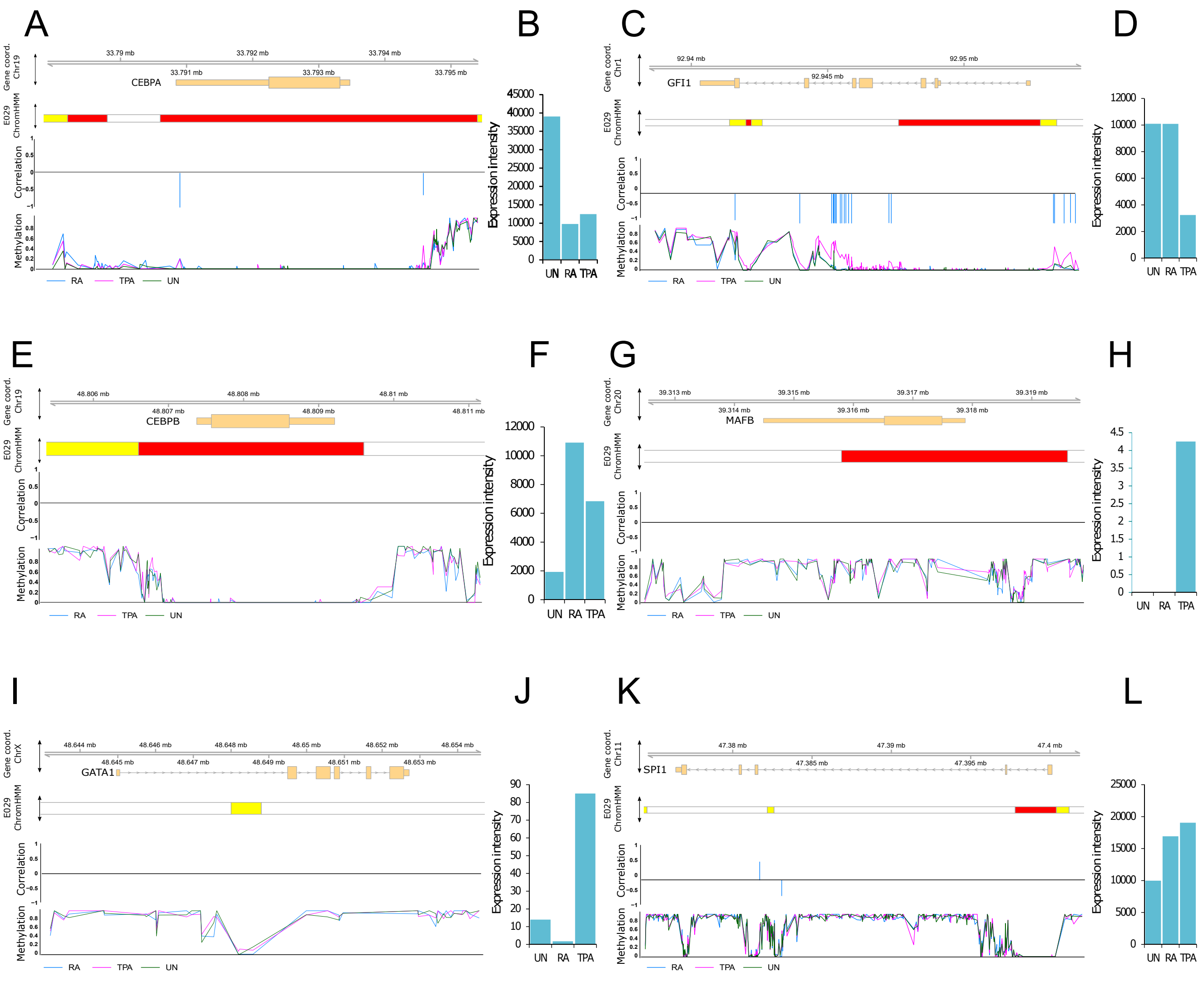

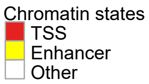

Figure S3

Figure S3: Key myeloid differentiation transcription factors are differentially methylated and expressed during expression. DNA methylation landscape and gene expression of transcription factors know to play important role in myeloid differentiation. Gene expression levels for the three differentiation states as shown in the blue bar plots correspond with the methylation and correlation profiles on their left. Tracks (from top to bottom): genomic coordinates; gene model; simplified chromatin segmentation based on E029 (primary monocyte cells from peripheral blood) from the ROADMAP epigenome project; bar plots depicted correlations of DMPs with differential gene expression (where a bar below 0 indicates negative correlation and a bar above 0 indicates positive correlation); line plot of DNA methylation rate of CpGs in UN, RA and TPA cells. A more detailed explanation of the tracks can be found in the legend of figure 4A.

Biology Open • Supplementary information

Supplementary tables

	**UN**	**RA**	**TPA**
QC-passed reads	1,075,185,936	1,070,133,636	1,096,718,018
Read pairs	453,160,937	453,160,937	433,631,362
Unpaired reads	37,984,963	37,984,963	36,188,487
Unmapped reads (%)	12	10	18
Duplicates (%)	4	4	4
Genome-wide coverage(x)	28.87	29.43	27.56
CpGs identified	26681926	26699651	26647233
CpG coverage	21.9	22.6	20.2
ChrM conversion	0.998761	0.999071	0.998135

Table S1: Read and alignment statistics of the whole genome bisulphite sequencing data used in this study.

Tables S2-S13: enrichment of GO molecular function terms in modules M1-M12

Click here to Download Table S2-S13

Table S14: enrichment of GO biological process terms in module M6

Click here to Download Table S14

Biology Open • Supplementary information
